# Reuse of Immobilized *Weissella cibaria* PN3 for Long-Term Production of Both Extracellular and Cell-Bound Glycolipid Biosurfactants

**DOI:** 10.3389/fbioe.2020.00751

**Published:** 2020-07-03

**Authors:** Tipsuda Subsanguan, Nichakorn Khondee, Parisarin Nawavimarn, Witchaya Rongsayamanont, Chien-Yen Chen, Ekawan Luepromchai

**Affiliations:** ^1^International Program in Hazardous Substance and Environmental Management, Graduate School, Chulalongkorn University, Bangkok, Thailand; ^2^Research Program on Remediation Technologies for Petroleum Contamination, Center of Excellence on Hazardous Substance Management, Chulalongkorn University, Bangkok, Thailand; ^3^Department of Natural Resources and Environment, Faculty of Agriculture Natural Resources and Environment, Naresuan University, Phitsanulok, Thailand; ^4^Microbial Technology for Marine Pollution Treatment Research Unit, Department of Microbiology, Faculty of Science, Chulalongkorn University, Bangkok, Thailand; ^5^Faculty of Environment and Resource Studies, Mahidol University, Nakhon Pathom, Thailand; ^6^Department of Earth and Environmental Sciences, National Chung Cheng University, Chiayi, Taiwan

**Keywords:** glycolipids, immobilization, lactic acid bacteria, biosurfactant production, biosurfactant characterization

## Abstract

Lactic acid bacteria (LABs) are generally recognized as safe (GRAS), and therefore, LAB biosurfactants are beneficial with negligible negative impacts. This study aims to maintain the biosurfactant producing activity of an LAB strain, *Weissella cibaria* PN3, by immobilizing the bacterial cells on a commercial porous carrier. For biosurfactant production, 2% soybean oil was used as the carbon source. After 72 h, immobilized cells were reused by replacing production medium. The extracellular and cell-bound biosurfactants were extracted from the resulting cell-free broth and cell pellets, respectively. SEM images of used immobilizing carriers showed increased surface roughness and clogged pores over time. Thus, the immobilizing carriers were washed in PBS buffer (pH 8.0) before reuse. To maintain biosurfactant production activity, immobilized cells were reactivated every three production cycles by incubating the washed immobilizing carriers in LB medium for 48 h. The maximum yields of purified extracellular (1.46 g/L) and cell-bound biosurfactants (1.99 g/L) were achieved in the 4th production cycle. The repeated biosurfactant production of nine cycles were completed within 1 month, while only 2 g of immobilized cells/L were applied. The extracellular and cell-bound biosurfactants had comparable surface tensions (31 – 33 mN/m); however, their CMC values were different (1.6 and 3.2 g/L, respectively). Both biosurfactants had moderate oil displacement efficiency with crude oil samples but formed emulsions well with gasoline, diesel, and lavender, lemongrass and coconut oils. The results suggested that the biosurfactants were relatively hydrophilic. In addition, the mixing of both biosurfactants showed a synergistic effect, as seen from the increased emulsifying activity with palm, soybean and crude oils. The biosurfactants at 10 – 16 mg/mL showed antimicrobial activity toward some bacteria and yeast but not filamentous fungi. The molecular structures of these biosurfactants were characterized by FTIR as different glycolipid congeners. The biosurfactant production process by immobilized *Weissella cibaria* PN3 cells was relatively cheap given that two types of biosurfactants were simultaneously produced and no new inoculum was required. The acquired glycolipid biosurfactants have high potential to be used separately or as mixed biosurfactants in various products, such as cleaning agents, food-grade emulsifiers and cosmetic products.

## Introduction

Surfactants are amphiphilic molecules with a wide range of applications, such as cleaning, wetting, dispersing, emulsifying, and foaming. Surfactants are mainly synthesized from petroleum, leading to growing concerns over their sustainable production as well as their potential toxicity and persistence. The alternative is producing biosurfactants from various microorganisms, especially the genera *Pseudomonas*, *Bacillus*, *Acinetobacter*, *Gordonia*, and *Candida* (Mnif and Ghribi, 2016). This study focuses on lactic acid bacteria (LAB), which are generally recognized as safe (GRAS). It is expected that biosurfactants extracted from LABs are beneficial with negligible negative impacts. Moreover, the scale-up of biosurfactant production using GRAS will require simple microbiological practices and instruments. Some LABs such as *Lactobacillus brevis*, *Lactobacillus paracasei, Lactobacillus plantarum*, and *Lactococcus lactis* produce extracellular and cell-bound biosurfactants with different properties simultaneously (Cornea et al., 2016; Souza et al., 2017; Vecino et al., 2017). Thus, the production of two biosurfactants by using an LAB strain will be relatively cost-effective and convenient.

Biosurfactants from LABs have good surface activity, emulsification activity, antimicrobial activities, and antiadhesive activities (Satpute et al., 2016; Vecino et al., 2018). The biosurfactants from LABs have been classified into several groups, such as glycolipopeptide from *Lactobacillus pentosus* (Vecino et al., 2015), glycolipid from *Lactobacillus helveticus* MRTL91 (Sharma et al., 2014), glycoprotein from *L. plantarum* (Madhu and Prapulla, 2014) and lipoprotein from *Pediococcus dextrinicus* SHU1593 (Ghasemi et al., 2019). The commercial application of LAB biosurfactants is limited by the low biosurfactant yield and inadequate information on their structural composition and functional characteristics (Bustos et al., 2018). The biosurfactant yields from LABs are usually in the range of mg per liter (Sharma et al., 2014, 2015). To increase biosurfactant yields, specific LAB strains are selected and cultivated in an optimized medium. For example, *L. pentosus* CECT-4023 produced biosurfactant at 1.7 g/L with whey medium as carbon source, which is higher than that from other LABs strains in whey medium (Rodrigues et al., 2006). *Lactobacillus delbrueckii* N2 produced biosurfactant at 3.03 g/L and 2.77 g/L with molasses and glycerol as the carbon sources, respectively (Mouafo et al., 2018).

Another approach for increasing total biosurfactant yields is to reuse the bacterial biomass in sequential fermentation. Bustos et al. (2018) found that *L. pentosus* cells can be subjected to 3 fermentation cycles after extracting the cell-bound biosurfactants with phosphate buffered saline. In addition, the rhamnolipid yield of *Achromobacter* sp. PS1 increased 258% after 5 sequential cycles of a fill-and-draw operation (Joy et al., 2019). To facilitate the reuse of bacterial inoculum and increase cell density, the bacteria may be immobilized on a solid support. For example, polyethylene oxide (PEO)-immobilized *Pseudomonas aeruginosa* BN10 is quite stable and can be reused in semicontinuous rhamnolipid production for nine cycles (Christovaa et al., 2013). However, there are some disadvantages of cell immobilization over long-term usage, such as cell inactivation during the process, mass transfer limitations, accumulation of toxic metabolites or inhibitor products inside the carrier, and cell leakage due to uncontrolled cell growth in the blocked area (Partovinia and Rasekh, 2018). This study therefore investigated the effect of cell-washing solutions and reactivation processes on immobilized LABs.

This study used *Weissella cibaria* PN3, a local biosurfactant-producing LAB, as a model strain. The genera *Weissella* contains heterofermentative LABs that produce lactate, CO_2_, ethanol, or acetate from glucose, exploiting the phosphoketolase pathway (Hajfarajollah et al., 2018). *Weissella cibaria* has been shown to have high probiotic potential and produce various novel, non-digestible oligosaccharides and extracellular polysaccharides (Fusco et al., 2015). To improve biosurfactant production efficiency, in this study, this bacterium was immobilized on a commercial porous carrier using a cell attachment approach. After biosurfactant production, the immobilized cells were reused, and the extracellular and cell-bound biosurfactants were extracted from the resulting cell-free broth and cell pellets, respectively. The cell-washing and reactivating processes were investigated and performed to maintain the activity of reused cells. Finally, the biosurfactants were characterized for surface properties, molecular structure and antimicrobial activity. It is expected that the use of immobilized LAB cells will allow for the economical production of two different biosurfactants that can be applied in different products.

## Materials and Methods

### Biosurfactant-Producing Bacteria, Media, Porous Carriers and Chemicals

*Weissella cibaria* PN3 was isolated from traditional rice sausage in De Man Rogosa, and Sharpe (MRS) broth, which is composed of 10 g/L peptone, 8 g/L meat extract, 4 g/L yeast extract, 20 g/L D(+)-Glucose, 2 g/L K_2_HPO_4_, 5 g/L C_2_H_9_NaO_5_, 2.0 g/L C_16_H_17_N_3_O_7,_ 0.2 MgSO_4._7H_2_O and 0.05 g/L MnSO_4_.H_2_O. This bacterial strain has been found to be an effective biosurfactant producer with soybean oil as a substrate. It was deposited as MSCU 0840 at the MSCU culture collection in the Department of Microbiology, Faculty of Science, Chulalongkorn University, which is a partnership with the Thailand Bioresource Research Center (TBRC). MRS medium was used to maintain the bacterium under aerobic conditions at room temperature (28–30°C). Although most *Weissella* bacteria are facultatively anaerobic chemoorganotrophs (Fusco et al., 2015), Papagianni (2012) reported that *Weissella paramesenteroides* DX produced the highest biomass under fully aerobic conditions. Our previous study also showed that aerobic cultivation enhanced the growth of *Weissella cibaria* PN3. A commercial sponge form carrier, Aquaporousgel (Nisshinbo Chemical Inc. Tokyo, Japan), was used for bacterial cell immobilization because of its high porosity, hydrophilic nature and stability. Aquaporousgel is composed of >80% polyurethane resin, <10% barium sulfate and <3% polyethylene polypropylene glycol and has an average dimension of 0.7 × 0.7 cm (Supplementary Figure 1). All other chemicals were of analytical grade and purchased from Sigma-Aldrich Co., LLC.

### Bacterial Immobilization Process

To prepare bacterial inoculum, bacterial colonies from MRS agar plates were inoculated in Luria-Bertani (LB) medium and cultivated on a rotary shaker at 200 rpm until an OD_600_ of 1.0 was reached. LB medium was used instead of MRS broth because it is cheaper and contains sufficient nutrients to support bacterial growth. The bacterial cells were immobilized on the surface of the carrier using the attachment method. Briefly, 10% (v/v) bacterial inoculum was added to 100 mL of LB medium containing 1.8% (w/v) porous carrier. The conditions provided good mixing of submerged carriers in the medium (Supplementary Figure 2A). The flasks were incubated under shaking conditions for 2 days, of which the numbers of attached bacteria were the highest at 2.2 × 10^9^ CFU/g immobilized cells (Supplementary Figure 1C). To measure the bacterial number, the immobilizing carrier was cut into small pieces, rehydrated in 0.85% (w/v) NaCl solution and sonicated in an ultrasonicated bath for 2 min to dislodge the cells. The detached bacterial cells were counted by the drop plate technique using 25% (v/v) LB agar. In addition, micrographs of immobilized cells were recorded using a Scanning Electron Microscope and Energy Dispersive X-ray Spectrometer – SEM-EDS (IT500HR) at the Scientific and Technological Research Equipment Centre (STREC), Chulalongkorn University, Thailand.

### Biosurfactant Production

The immobilized cells were used for biosurfactant production in batch mode. After the immobilization process, the immobilized medium was replaced with 100 mL of basal medium composed of 5 g/L glucose, 0.1 g/L yeast extract, 10.17 g/L NaNO_3_, 1 g/L K_2_HPO_4_, 0.5 g/L KH_2_PO_4_, 0.1 g/L KCl, 5 g/L MgSO_4_.7H_2_O, 0.01 g/L CaCl_2_, 0.06 g/L FeSO_4_.7H_2_O, 0.326 g/L MnSO_4_.H_2_O and 0.005 (%v/v) trace elements (0.26 g/L H_3_BO_3,_ 0.5 g/L CuSO_4_.H_2_O, 0.5 g/L MnSO_4_.H_2_O, 0.06 g/L MoNa_2_O_4_.2H_2_O, and ZnSo_4_.7H_2_O). Glucose serves as an inducer for supporting bacterial growth and promoting biosurfactant production (Nurfarahin et al., 2018). Soybean oil at 2% (v/v) was added as a substrate for biosurfactant production following Laorrattanasak et al. (2016). After incubating the immobilized cells for 3 days, the culture medium was poured out of the flask for biosurfactant recovery, while the immobilized cells were maintained inside for another production cycle. New production medium was added, and the cells were incubated as mentioned earlier. In the initial experiment, the immobilized cells were reused for several production cycles without the cell-washing and reactivating processes. The biosurfactant-producing activity of immobilized cells was determined based on crude biosurfactant yields as well as the numbers of bacteria remaining on the immobilizing carriers (immobilized cells) and washed-out bacteria (suspended cells). All experiments were analyzed statistically via two-way ANOVA followed by Tukey’s multiple comparison test (*p* < 0.05) in GraphPad Prism 8.0.1.

### Cell-Washing and Reactivating Processes

During biosurfactant production, the immobilizing carriers might become clogged by residual soybean oil and bacterial metabolites, which could decrease cell growth and biosurfactant yield in the following production cycle. Therefore, the influences of cell washing and reactivation processes were investigated. The immobilized cells were washed after removing the culture medium by adding 100 mL of washing solutions to the flask and shaking at 200 rpm for 10 min. The tested washing solutions included 5–15% (w/v) NaCl (Kebbouche-Gana et al., 2013) and phosphate buffered saline (PBS), pH 8 (10 mM KH_2_PO_4_/K_2_HPO_4_ with 150 mM NaCl) (Gudina et al., 2015). PBS is a general cell washing solution, while NaCl is an electrolyte that can enhance the detergency of biosurfactant residues on immobilizing carriers during washing. Concentration of NaCl was varied because increase in salinity enhances interaction between surfactant and oil while inhibiting interaction between surfactant and water (Nguyen and Sabatini, 2011). The washed immobilizing carriers were reused for biosurfactant production. The efficiency of the washing solution was determined from the biosurfactant producing activity of reused immobilized cells. When the biosurfactant producing activity of the reused immobilized cells decreased, the cells were reactivated by incubation in LB broth for 48 h. LB broth was selected because it is a rich medium that can rapidly promote bacterial growth and activity.

### Biosurfactant Solvent-Extraction Process and Characterization

The biosurfactants were recovered from the culture medium; solubilized extracellular biosurfactant is present in the supernatant and cell-bound biosurfactant is present in the suspended cell fraction. Initially, the culture medium was centrifuged at 8000 rpm for 10 min to separate cell pellets from the supernatant. The cell pellets were washed with 0.85% NaCl, while the supernatant was extracted with 10% (v/v) hexane to remove residual oil. The cell-bound biosurfactant was recovered by resuspending the cell pellets in methanol with shaking for 1 h. Although, most researchers use PBS to obtain cell-bound biosurfactants from cell pellets (Bustos et al., 2018), our preliminary results showed that almost all the biosurfactant in the cell pellet fraction could be extracted with methanol, while PBS (at pH 7.0 and 8.0) and chloroform gave lower yields (Supplementary Figure 3). The hydrophilicity of *Weissella cibaria* PN3 cell-bound biosurfactant was probably different from that of other LAB biosurfactants.

The cell-bound biosurfactant in methanol was extracted by an acid precipitation and solvent extraction method similar to the extraction method for extracellular biosurfactant in the supernatant. Briefly, the pH of the sample was adjusted to 2.0 with 6 M HCl to reduce the biosurfactant solubility before adding an equal volume of a chloroform and methanol mixture (2:1 v/v), and the solution was incubated in a rotary shaker at 200 rpm for 1 h (Khondee et al., 2015). The organic solvent was separated and evaporated by rotary evaporation. The viscous yellowish product was dissolved in methanol and filtered. The amount of crude biosurfactant was measured by weighing, while the crude biosurfactant yield was calculated as g/L based on the volume of the production medium. In this study, biosurfactant was not extracted from immobilized cells because residual oil and other bacterial metabolites accumulated on the carriers could be co-extracted with the biosurfactant, resulting in a product with high impurities.

Prior to characterization, the extracted biosurfactants were separated from impurities such as proteins and fatty acids following micelle-destabilization and ultrafiltration methods modified from Witek-Krowiak et al. (2011). Crude extracts of extracellular and cell-bound biosurfactants were dissolved in methanol to break up the micelles and diluted to 1.0 and 2.0 g/L, respectively. Concentrations lower than their critical micelle concentrations (CMCs) (Table 1) were selected to prevent the aggregation of biosurfactant molecules. Methanol solution containing biosurfactant monomers was filtered through a 5 kDa MWCO ultrafiltration membrane (Hydrosart Vivaflow200, Sartorius, United Kingdom). The biosurfactant monomers passed through the membrane into the permeate, while impurities were retained in the retentate. Methanol was removed from the permeate by evaporation at 40°C. After purification, biosurfactant weight decreased by 10–20%, while purified extracellular and cell-bound biosurfactant concentrations in PBS (pH 8.0) decreased to 0.8 and 1.9 g/L, respectively. The chemical composition of the purified biosurfactants were analyzed by the colorimetric method following Khondee et al. (2015). Total lipids, proteins and sugars were determined by sulfo-phospho-vanillin, Bradford assay and phenol-sulfuric acid, respectively. The functional groups of the purified biosurfactants were analyzed by Fourier transform infrared (FTIR) spectroscopy in ATR mode (Spectrum, GX, Perkin Elmer) at wavenumbers ranging from 4000 to 400 cm^–1^ and a resolution of 0.3 cm^–1^.

**TABLE 1:** Comparison of the yield and characteristics of biosurfactants from *Weissella cibaria* PN3 with those from other LAB strains in a single production cycle.

**LAB strains**	Maximum yield (g/L)*	**Lowest surface tension (mN/m)**	**CMC (g/L)**	Composition (%) Lipid: Sugar: Protein*	**Molecular Structure**	**References**
*Weissella cibaria* PN3						This study
Extracellular BS	1.46	31.3	1.6	48:31:10	Glycolipid	
Cell-bound BS	1.99	32.6	3.2	50:39:2	Glycolipid	
*Pediococcus dextrinicus* SHU1593 (Cell-bound BS)	–	39.0	2.7	52:1:57	Lipoprotein	Ghasemi et al., 2019
*Lactobacillus paracasei* (Extracellular BS)	–	25.0	1.4	25:6:21	Glycolipopeptide	Ferreira et al., 2017
*Lactobacillus agilis* CCUG31450 (Cell-bound BS)	0.84	42.5	7.5	–	Glycoprotein	Gudina et al., 2015
*Enterococcus faecium* MRTL9 (Cell-bound BS)	–	40.2	2.3	–	Glycolipid	Sharma et al., 2015
*Lactobacilllus helveticus* MRTL 91	0.80	39.5	2.5	–	Xylolipid	Sharma et al., 2014
*Lactobacillus pentosus* CECT-4023 (Extracellular BS)	1.80	39.5	–	–	Surfactin	Rodrigues et al., 2006
*Lactobacillus delbrueckii* N2 (Extracellular BS)	3.03	41.9	–	–	–	Mouafo et al., 2018

*The maximum yields and composition of biosurfactants were determined after biosurfactant purification by ultrafiltration. The maximum yields reported are from the 4th production cycle.

### Biosurfactant Property

As crude biosurfactants are more practical for commercial applications than expensive purified biosurfactants, biosurfactant properties of the crude biosurfactants were determined. Surface tension (ST) of the biosurfactants was measured using a digital tensiometer (Kruss, K10ST, Germany) at 25°C using the plate method. Critical micelle concentration (CMC) was determined from a plot of surface tension versus biosurfactant concentrations (Supplementary Figure 5 and Supplementary Table 1). Other surface activities were determined with various oil samples, including Bongkot light crude oil (BKC), Arab light/Arab extra light blend (ARL/AXL blend), gasoline, diesel, hydrocarbons, essential oils and vegetable oils. The crude oils were obtained from Thai Oil PCL, while other oil samples were purchased from local distributors. The biosurfactant samples were prepared by dissolving crude extract in PBS (pH 8.0) to yield a concentration that is four times the respective CMC (4xCMC). Concentrated biosurfactant solutions were used to ensure that the tested system contains sufficient biosurfactant molecules. To prepare mixed biosurfactants, 4xCMC extracellular and cell-bound biosurfactant solutions were mixed at a ratio of 1:1 (v/v). Sodium dihexyl sulfosuccinate (SDHS), a chemical surfactant, was used as control at a concentration of 0.4 g/L (4xCMC). All tests were conducted in triplicates.

The oil displacement activity was determined following Khondee et al. (2015). Briefly, 10 μL of biosurfactant was dropped onto the surface of the oil layer, which was formed by adding 20 μL of oil sample to 20 mL of distilled water in an 80-mm diameter petri dish. The study investigated the oil displacement activity in distilled water to find the potential application of biosurfactants in freshwater and wastewater. The diameter of the clear zone on the oil surface was measured to calculate the oil displacement efficiency using the following equation:

$$\mathrm{Oil}\mathrm{displacement}\left(\%\right)=\frac{\mathrm{Diameter}\,\mathrm{of}\,\mathrm{clear}\,\mathrm{zone}\times 100}{\mathrm{Diameter}\,\mathrm{of}\,\mathrm{water}\,\mathrm{surface}}$$

For emulsion formations, 2 mL each of biosurfactant and oil samples were added into 15 mL glass tubes, which were covered with caps. The glass tubes were shaken with vortexing for 1 min and left without disturbance for 24 h. The percentages of emulsion volume (EV) were calculated by the following equation:

$$\mathrm{Emulsion}\mathrm{Volume}\left(\%\right)=\frac{\text{Emulsion}\,\text{height},\mathrm{mm}\times \mathrm{Cross}\,\text{section}\,\text{area},{\text{mm}}^{2}}{\text{Total}\,\text{liquid}\,\text{volume},{\text{mm}}^{3}}$$

The antimicrobial activities of biosurfactants were determined by measuring the minimum inhibitory concentration (MIC) and minimum bactericidal concentration (MBC) on 96-well plates following a modified broth microdilution assay from Elshikh et al. (2016) at the Microbial Technology Service Centre, Department of Microbiology, Chulalongkorn University. The tested microorganisms were *Staphylococcus aureus*, *Escherichia coli, Candida albicans* and *Aspergillus niger*, which represented gram (+) bacteria, gram (−) bacteria, yeast and fungi, respectively.

## Results

### Initial Production of Biosurfactant by Immobilized Bacteria

**FIGURE 1:**
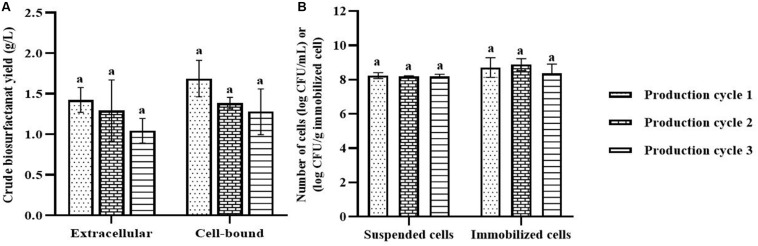
Crude biosurfactant yields **(A)** and bacterial cell numbers **(B)** after each production cycle without washing process in the 3rd production cycle. The biosurfactant yield was based on volume of production medium. The yields of extracellular/cell-bound biosurfactants were compared between production cycles. Error bars represent mean ± standard deviation (*n* = 3). Two-way ANOVA: Tukey’s Multiple Comparison Test was used for statically analysis. Different letters represent the significant value (*P* < 0.05).

**FIGURE 2:**
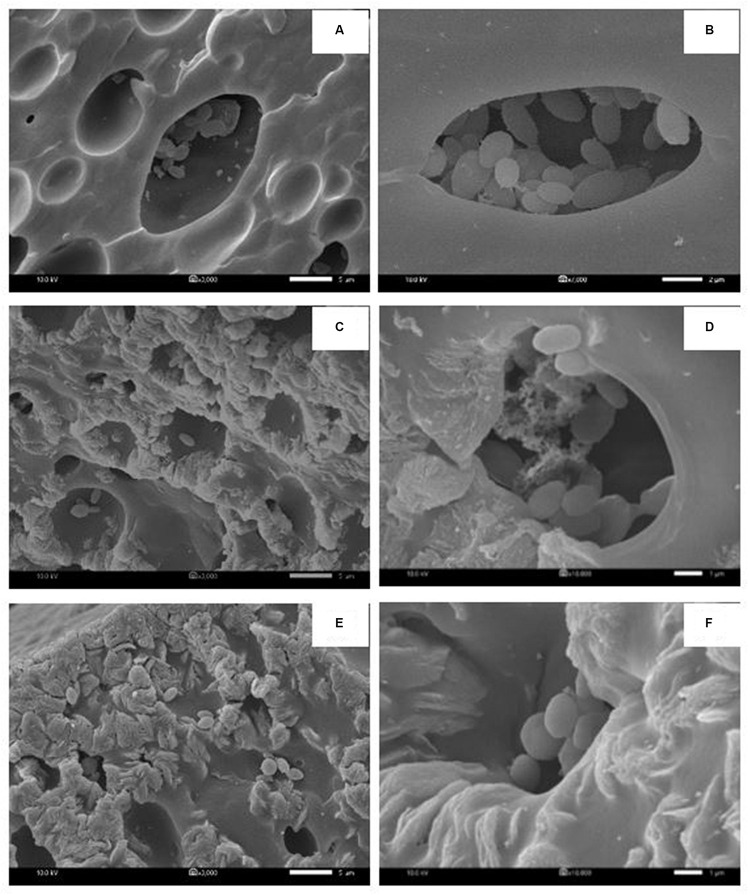
Characteristic of immobilized cells before biosurfactant production **(A,B)**, after the 1st production cycle **(C,D)** and after the 3rd production cycle **(E,F)**. The 3000X magnified image is shown on the left while the 10000X magnified image is displayed on the right. The immobilized cells from the 1st and 2nd biosurfactant production cycles were reused without washing process.

In each biosurfactant production cycle, the immobilized *Weissella cibaria* PN3 cells gradually produced biosurfactants as seen from the changes in the medium color from light yellow to milky white (Supplementary Figures 2B,C). The immobilized cells simultaneously produced an average of 1.25 and 1.44 g/L crude extracellular and cell-bound biosurfactants, respectively (Figure 1A). After biosurfactant production, the color of the porous carrier changed from white to yellow (Supplementary Figure 1). The pH values of the culture medium were reduced from 7 to an average of 6.5 after biosurfactant production, which was probably due to the production of acidic metabolites such as lactic acid (Fusco et al., 2015). During biosurfactant production, bacterial cells were grown from the immobilizing carriers, as the number of suspended cells was 1.8 × 10^8^ CFU/mL, while the number of immobilized cells was unchanged at 7.7 × 10^8^ CFU/g immobilized cells. The immobilized cells could be reused in new production medium without any washing process for at least three cycles. The results indicated that the immobilized bacteria had high biosurfactant producing activity. The crude extracellular and cell-bound biosurfactant yields were not significantly different (*p* < 0.05) between production cycles (Figure 1A). Nonetheless, there was a decreasing trend of both biosurfactants from cycle 1 to 3. The decrease in the biosurfactant yields was not related to bacterial growth because the numbers of immobilized and suspended cells at the end of each cycle were maintained at approximately 7.3 × 10^8^ CFU/g and 1.6 × 10^8^ CFU/mL, respectively (Figure 1B). The characteristics of immobilizing carriers before and after biosurfactant production were therefore compared using SEM analysis. After immobilization, many cells were attached inside the pores of the carrier, and the surface of the carrier was mostly clean and smooth (Figures 2A,B). However, the immobilizing carriers had clogged pores and increased surface roughness after biosurfactant production in cycle 1. The immobilizing carriers after three production cycles had the thickest biofilm layers (Figures 2C–F), which corresponded with the decreasing trend of biosurfactant yields (Figure 1A).

### Effect of Cell-Washing Solution on Biosurfactant Production

**FIGURE 3:**
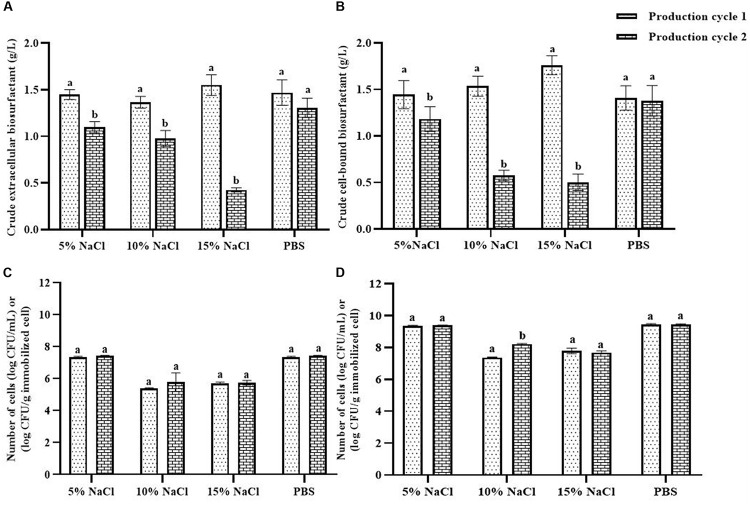
Comparison of extracellular biosurfactant yield **(A)** and cell-bound biosurfactant yield **(B)** observed with different washing solutions after the 1st production cycle. The immobilized cells were washed with either sodium chloride (NaCl) solution at 5, 10, and 15% or phosphate buffer saline (PBS) solution, pH 8.0 before reused in the 2nd production cycle. The numbers of suspended **(C)** and immobilized cells **(D)** were monitored after each production cycle. The biosurfactant yield was based on volume of production medium. The yields of extracellular/cell-bound biosurfactants were compared between production cycles. Error bars represent mean ± standard deviation (*n* = 3). Two-way ANOVA: Sidak’s Multiple Comparison Test was used for statically analysis. Different letters represent the significant value (*P* < 0.05).

NaCl and PBS solutions were compared for cleaning the immobilizing carriers after being used for 1 cycle (i.e., Production cycle 1). The biosurfactant production activity of PBS-washed immobilizing carriers was the same as that of production cycle 1, while the biosurfactant yields of NaCl-washed immobilizing carriers decreased with increasing NaCl concentrations (Figure 3A). In addition, PBS-washed immobilizing carriers had higher biosurfactant yields than NaCl-washed immobilizing carriers (Figure 3A). The ranges of crude extracellular and cell-bound biosurfactant yields after the PBS cell-washing process from 2 cycles were 1.22–1.62 and 1.21–1.54 g/L, respectively (Figures 3A,B). Therefore, washing immobilized cells with PBS did not influence biosurfactant yield. The cell numbers on immobilizing carriers washed with PBS and 5% NaCl were 2.8 × 10^9^ and 2.3 × 10^9^ CFU/g immobilized cells, respectively, which were similar to those of the unwashed carriers (Figure 1B). On the other hand, 10 and 15% NaCl solutions decreased the cell numbers to 2.4 × 10^7^ and 6.5 × 10^7^ CFU/g immobilized cells, respectively (Figures 3C,D), which corresponded with the decreasing biosurfactant yields (Figures 3A,B). The PBS washing solution contained negligible bacterial cells but had biosurfactant in the range of 0.05 to 0.09 g/L, whereas 5–15% (w/v) NaCl washing solution contained 0.02–0.03 g/L biosurfactant. The concentrations of biosurfactant in all washing solutions were quite low, so they were discarded. In addition to washing the biosurfactant residues from the immobilizing carriers, PBS solutions also cleaned biofilm matrices. The SEM images of PBS-washed immobilizing carriers showed distinct bacterial cells on the surface (Figures 5A,B) with less biofilm than the unwashed carrier (Figures 2C,D). Therefore, PBS, pH 8.0, was selected as the solution in the cell washing process to remove metabolites covering the carrier in each production cycle.

### Effect of Washing and Reactivation Processes on Long-Term Biosurfactant Production

**FIGURE 4:**
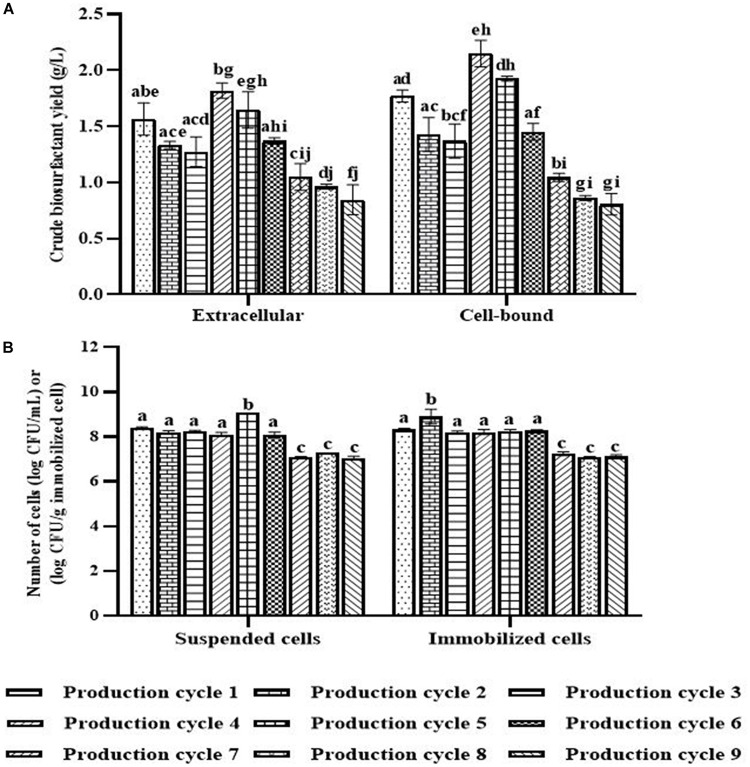
Crude biosurfactant yields **(A)** and bacterial cell numbers **(B)** after each production cycle with washing and reactivation processes. The immobilized cells were washed with PBS at pH 8.0 before reused in the next cycle. Before the 4th and 7th production, the washed immobilized bacteria were reactivated with Luria-Bertani (LB) for 48 h. The biosurfactant yield was based on volume of production medium. The yields of extracellular/cell-bound biosurfactants were compared between production cycles. Error bars represent mean ± standard deviation (*n* = 3). Two-way ANOVA: Tukey’s Multiple Comparison Test was used for statically analysis. Different letters represent the significant value (*P* < 0.05).

**FIGURE 5:**
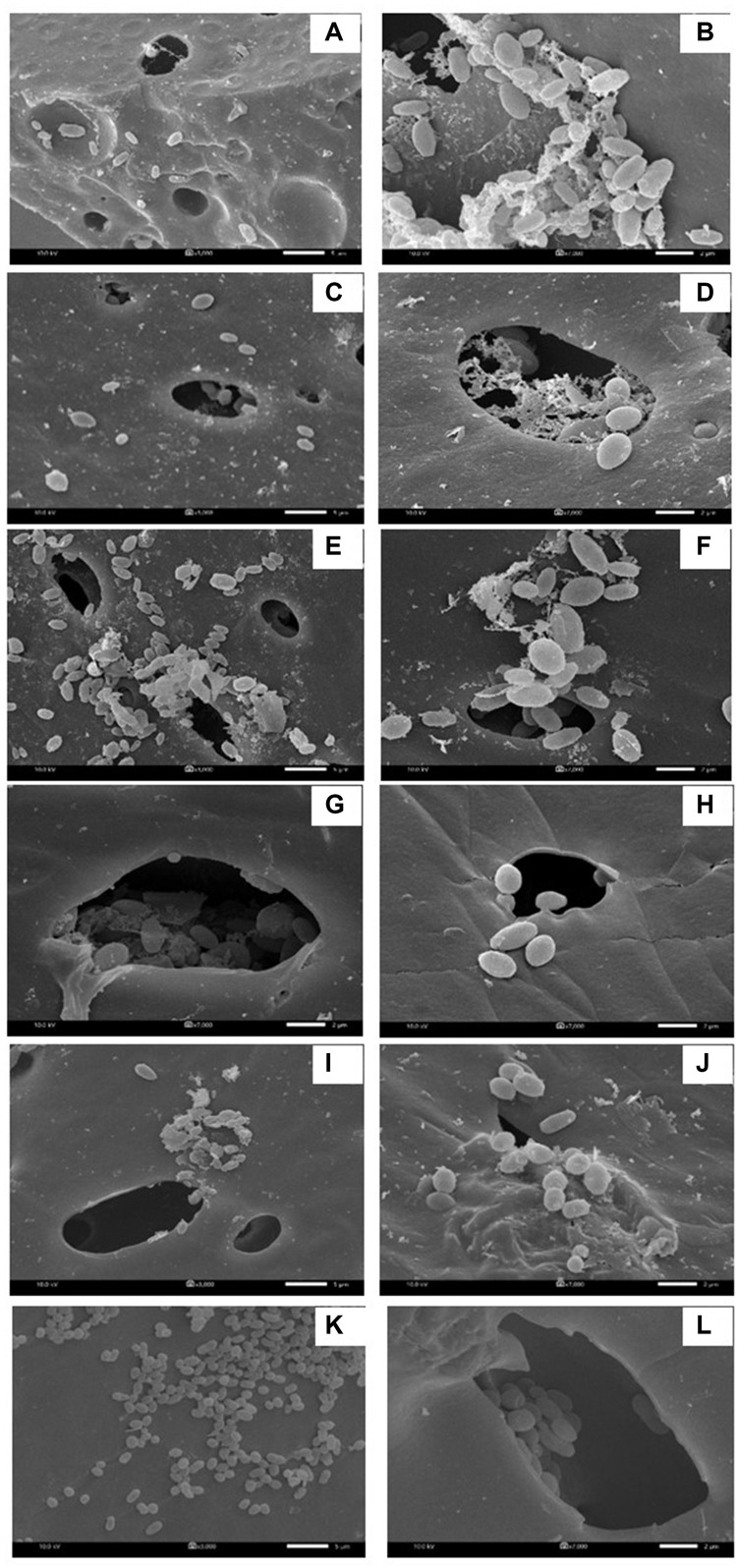
Characteristic of immobilized cells after washing and reactivation processes. There were washed immobilized cells from the 1st production cycle **(A,B)**, 3rd production cycle **(C,D)**, 4th production cycle after cell reactivation **(E,F)**, 6th production cycle **(G,H)**, 7th production cycle after cell activation **(I,J)** and 9th production cycle **(K,L)**. The 3000X magnified image is shown on the left while the 7000X magnified image is displayed on the right.

When the PBS-washed immobilizing carriers were reused for several production cycles (i.e., Productions cycle 2 and 3), the crude biosurfactant yields tended to decrease compared to the new immobilized bacteria (i.e., Production cycle 1) (Figure 4). This study therefore investigated cell reactivation by adding LB medium to the washed immobilizing carriers at every three production cycles. After reactivation, the yields of crude extracellular and cell-bound biosurfactants were significantly increased (*p* < 0.05), and the highest yields were found in the 4th cycle at 1.82 and 2.15 g/L, respectively (Figure 4A). The numbers of immobilized and suspended cells after cell reactivation were 1.7 × 10^8^ CFU/g and 1.3 × 10^8^ CFU/mL, respectively, which were not significantly different (*p* < 0.05) from those of the previous cycles (Figure 4B). However, the SEM images of the reactivated immobilizing carriers in the 4th cycle showed more bacterial cells on the carrier surface (Figures 5E,F) than that of the washed immobilizing carriers (Figures 5C,D). Therefore, the reactivation process probably allowed the bacteria to be more active but not enough for growth. The immobilizing carriers showed only few bacterial cells on the surface after using in cycle 6th (Figures 5G,H). After the second round of reactivation, there were less bacterial cells on immobilizing carriers in cycle 7th (Figures 5I,J) than those in cycle 4th.

In the 7th to 9th production cycles, the yields of crude extracellular and cell-bound biosurfactants were significantly decreased from the previous cycles (*p* < 0.05) to 0.85 and 0.81 g/L, respectively (Figure 4A). The results corresponded with the significantly decreased numbers of immobilized and suspended cells to 1.4 × 10^7^ CFU/g immobilized cells and 1.2 × 10^7^ CFU/mL, respectively (Figure 4B). The SEM images of immobilizing carriers in the 9th cycle also showed varied clean surfaces without any biofilm residues, and most bacterial cells were inside the pores and might have low access to nutrients (Figures 5K,L). Consequently, the immobilizing carriers should not be reused after nine cycles. The total yields of crude extracellular and cell-bound biosurfactants from these nine production cycles were 11.86 and 12.81 g/L, respectively. All experiments were carried out in a 1-month period, and the immobilizing carrier was applied at only 2 g immobilized cells/L accumulated production medium. The results indicated that the cell washing and reactivation processes were necessary for long-term biosurfactant production. The washing solutions cleaned the carrier surface, allowing interactions between bacterial cells, nutrients and oxygen. The reactivation of cells in LB medium should be performed when the washed immobilizing carriers are repeatedly used in several cycles or the production yield significantly decreases.

### Properties of Extracellular and Cell-Bound Biosurfactants

Crude extracellular and cell-bound biosurfactants obtained from every cycle were combined before analysis (Supplementary Figure 4). In addition, a mixture of extracellular and cell-bound biosurfactants at a 1:1 ratio was examined to simulate the activity of both biosurfactants during bacterial growth. The surface activities of extracellular and cell-bound biosurfactants are shown in Table 1 and Supplementary Figure 5. The extracellular and cell-bound biosurfactants had comparable surface tensions (31 – 33 mN/m); however, their CMC values were different (1.6 and 3.2 g/L, respectively; Table 1).

The capabilities of crude biosurfactants to emulsify petroleum oils, hydrocarbons, essential oils and vegetable oils were investigated as emulsion volume (EV) %. The highest EV (>70%) was found from extracellular biosurfactants with lavender, followed by gasoline, lemongrass, diesel oil, and coconut oil (Table 2). Extracellular and cell-bound biosurfactants did not form emulsions when mixed individually with palm and soybean oils, while mixture of both biosurfactants had an EV of 40% with palm and soybean oil. In the case of petroleum oils, mixed biosurfactants had slightly increased emulsifying activity compared to single biosurfactants. Consequently, there was a synergistic effect between extracellular and cell-bound biosurfactants for emulsifying certain oil types. It is thus possible to apply single or mixed biosurfactants as bioemulsifiers for various vegetable oils and essential oils used in food and cosmetic products.

**TABLE 2:** Oil displacement and emulsifying activities of crude extracellular, cell-bound, mixed biosurfactants (4xCMC), and synthetic surfactant (SDHS) on various oils.

**Oil types**	**Oil displacement (%)**				**Emulsion (%) after 1 days**			
	**Extracellular BS**	**Cell-bound BS**	**Mixed BS (1:1)**	**SDHS**	**Extracellular BS**	**Cell-bound BS**	**Mixed BS (1:1)**	**SDHS**
**Petroleum oils**								
BKC crude oil	68.4 ± 4.9	59.8 ± 2.9	44.6 ± 4.8	32.2 ± 3.8	0.0 ± 0.0	0.0 ± 0.0	33.3 ± 0.0	0.0 ± 0.0
ARL/AXL blend crude oil	38.0 ± 2.9	45.1 ± 4.1	22.8 ± 2.7	15.7 ± 4.1	0.0 ± 0.0	30.7 ± 4.6	18.7 ± 2.3	0.0 ± 0.0
Gasoline	0.0 ± 0.0	0.0 ± 0.0	0.0 ± 0.0	0.0 ± 0.0	82.7 ± 2.3	80.0 ± 4.0	86.7 ± 5.2	44.0 ± 4.0
Diesel	0.0 ± 0.0	0.0 ± 0.0	0.0 ± 0.0	0.0 ± 0.0	78.7 ± 2.3	69.3 ± 2.3	81.3 ± 2.3	48.0 ± 4.0
**Hydrocarbons**								
Hexane	0.0 ± 0.0	0.0 ± 0.0	0.0 ± 0.0	0.0 ± 0.0	0.0 ± 0.0	0.0 ± 0.0	0.0 ± 0.0	34.0 ± 5.3
Octane	100.0 ± 0.0	36.1 ± 4.8	89.9 ± 4.7	100.0 ± 0.0	0.0 ± 0.0	0.0 ± 0.0	0.0 ± 0.0	36.0 ± 2.8
Decane	84.0 ± 4.4	25.6 ± 2.6	44.1 ± 5.9	39.6 ± 5.5	0.0 ± 0.0	0.0 ± 0.0	0.0 ± 0.0	9.3 ± 2.5
Hexadecane	88.3 ± 4.2	19.6 ± 2.6	43.1 ± 4.2	66.7 ± 4.8	0.0 ± 0.0	0.0 ± 0.0	0.0 ± 0.0	0.0 ± 0.0
**Essential oils**								
Lavender	62.6 ± 2.8	18.0 ± 1.3	22.9 ± 3.2	17.3 ± 2.1	100.0 ± 0.0	100.0 ± 0.0	100.0 ± 0.0	100.0 ± 0.0
Lemongrass	55.0 ± 3.2	16.4 ± 2.4	27.1 ± 1.8	14.0 ± 2.3	80.0 ± 0.0	80.0 ± 0.0	80.0 ± 0.0	73.3 ± 2.3
Orange	0.0 ± 0.0	0.0 ± 0.0	0.0 ± 0.0	0.0 ± 0.0	0.0 ± 0.0	0.0 ± 0.0	0.0 ± 0.0	0.0 ± 0.0
**Vegetable oils**								
Coconut	0.0 ± 0.0	0.0 ± 0.0	0.0 ± 0.0	0.0 ± 0.0	74.7 ± 2.3	68.0 ± 4.0	57.3 ± 4.5	62.0 ± 2.8
Palm	46.5 ± 4.7	15.7 ± 1.1	34.0 ± 2.8	23.4 ± 2.6	0.0 ± 0.0	0.0 ± 0.0	40.0 ± 0.0	64.0 ± 5.7
Soybean	86.4 ± 3.3	34.1 ± 2.1	52.8 ± 2.6	70.4 ± 1.2	0.0 ± 0.0	0.0 ± 0.0	40.0 ± 0.0	52.0 ± 0.0

*Data are presented as mean ± standard deviation (*n* = 3).*

The emulsifying activities of all biosurfactant samples were comparable to that of the model synthetic surfactant, SDHS (Table 2). Similar to the emulsifying activity, the oil displacement activity of the extracellular biosurfactants was usually higher than those of the cell-bound and mixed biosurfactants, of which >80% oil displacement activities were from octane, decane, hexadecane and soybean oil (Table 2). The mixed biosurfactants showed a competitive effect on oil displacement activity, as seen from the decreased % oil displacement when compared to a single biosurfactant. Nonetheless, the biosurfactants had comparable oil displacement activities with SDHS, of which they could displace every tested oil sample except gasoline, diesel, hexane, orange oil and coconut oil, which were similar to SDHS. All biosurfactants had moderate oil displacement activity ranges of 23 – 68% with both BKC and ARL/AXL blend crude oil, and they should be formulated with other surfactants before use as oil dispersants.

The antimicrobial activity of crude biosurfactants derived from *Weissella cibaria* PN3 was determined by using *Staphylococcus aureus*, *Escherichia coli, Candida albicans* and *Aspergillus niger* (Table 3). The results showed that both biosurfactants showed antimicrobial activity toward some bacteria and yeast but not filamentous fungi. The extracellular biosurfactant had slightly lower antimicrobial activity than the cell-bound biosurfactant. The lowest concentration of cell-bound biosurfactant for killing *S. aureus* and *C. albicans* was 4.5x CMC (14.4 g/L), whereas extracellular biosurfactant had an MBC of 6.7x CMC (10.7 g/L) for *S. aureus* and *C. albicans* and 10x CMC (16.0 g/L) for *E. coli.*

**TABLE 3:** Minimum inhibitory concentration (MIC) and minimum bactericidal concentration (MBC) of crude extracellular and cell-bound biosurfactants on various microorganisms.

**Microorganisms**	**Extracellular BS**		**Cell-bound BS**	
	**MIC**	**MBC**	**MIC**	**MBC**
*Staphylococcus aureus*	6.7x CMC	6.7x CMC	3.0x CMC	4.5x CMC
*Escherichia coli*	6.7x CMC	10.0x CMC	4.5x CMC	6.7x CMC
*Candida albican*	3.0x CMC	6.7x CMC	2.0x CMC	4.5x CMC
*Aspergillus niger*	–	–	10.0x CMC	–

### Characterization of Extracellular and Cell-Bound Biosurfactants

**FIGURE 6:**
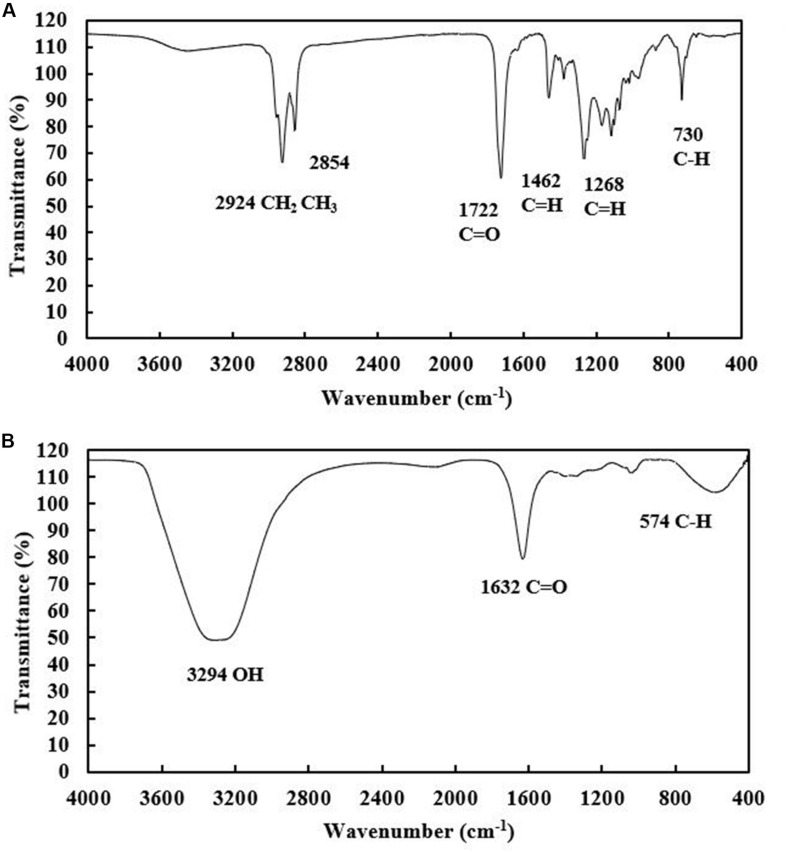
FTIR spectra of purified extracellular **(A)** and cell-bound biosurfactants **(B)** from *Weissella cibaria* PN3.

Both the extracellular and cell-bound biosurfactants were multicomponent molecules constituted by different concentrations of lipids, sugars and proteins. The lipid:sugar:protein ratios of purified extracellular and cell-bound biosurfactants were 48:31:10 and 50:39:2, respectively (Table 1). The FTIR spectra of purified extracellular and cell-bound biosurfactants revealed the composition of the polysaccharide and lipid fractions (Figure 6). The predominant adsorption bands in the FTIR spectrum of extracellular biosurfactant were observed at 2924 cm^–1^, 2854 cm^–1^ (CH stretching of CH2 CH3), 1722 cm^–1^ (C = O stretching of carboxyl group), 1462 cm^–1^ (C = H stretching), 1268 cm^–1^ (C-O stretching) and 730 cm^–1^ (C-H stretching), while the main adsorption bands of cell-bound biosurfactant were found at 3294 cm^–1^ (OH group), 1632 cm^–1^ (C = O stretching of carboxyl group) and 730 cm^–1^ (C-H stretching). The functional group and fingerprint regions from different LAB strains were compared (Sharma and Saharan, 2014; Antoniou et al., 2015; Sharma et al., 2015), and the results revealed that the extracellular and cell-bound biosurfactants were glycolipid biosurfactants with lipid hydrophobic chains and sugar hydrophilic parts. However, these biosurfactants are different glycolipid congeners. The FTIR spectra of crude biosurfactants (Supplementary Figure 6) confirm that ultrafiltration increases the purity of both extracellular and cell-bound biosurfactants.

## Discussion

This is the first experiment to use an immobilization technique to maintain the biosurfactant producing activity of an LAB strain. Immobilized *Weissella cibaria* PN3 cells simultaneously produced extracellular and cell-bound biosurfactants at an almost 1:1 ratio, and their crude biosurfactant yields were in the range of 1.25 – 2.15 g/L when soybean oil was used as a substrate. The purification of crude biosurfactants was carried out by ultrafiltration and the maximum yields of purified extracellular and cell-bound biosurfactants were 1.46 and 1.99 g/L, respectively (Table 1). In a single production cycle, the purified biosurfactant yields were comparable to those of other efficient LAB strains, such as *L. pentosus* CECT-4023 (Rodrigues et al., 2006) and *L. delbrueckii* N2 (Mouafo et al., 2018); however, total biosurfactant yields from *W. cibaria* PN3 (3.45 g/L) were the highest (Table 1). These results highlight the benefits of using LABs for simultaneous production of two biosurfactant types.

The immobilizing carriers were reused in new biosurfactant production cycles to reduce the cost of new inoculum preparation. However, the biosurfactant yields tended to decrease after three production cycles, which was probably due to changes in cell activity as well as biofilm formation on the carrier surface. This situation limited the interactions between media, oxygen and immobilized cells and led to the inhibition of biosurfactant production. To clean the immobilizing carriers, PBS, pH 8.0, was found to successfully remove biosurfactant residues and biofilm but not the attached cells. Residual soybean oil could be removed from the carrier surface by the reaction between alkali compounds in PBS and organic components in soybean oil to produce soap (Kurnia et al., 2020). However, cell toxicity was observed at high NaCl concentrations probably due to the detergency effect of NaCl-biosurfactant complexes (Nguyen and Sabatini, 2011) and/or the osmotic and specific ion effects of NaCl (Yan et al., 2015).

To further maintain biosurfactant producing activity, cell reactivation by adding fresh LB medium to the washed immobilizing carriers was investigated. The immobilized cells produced higher yields of extracellular and cell-bound biosurfactants in the following cycle. However, the bacterial numbers remained the same, which suggested that the reactivation process promoted biosurfactant production by activating cells and their enzymes for the subsequent cycle. Similarly, Suttinun et al. (2010) reported that the inoculation of *Rhodococcus* sp. L4-immobilized cumin seeds in fresh MS medium for 12–24 h after TCE degradation could reactivate the immobilized cells, which produced new enzymes involved in TCE degradation. Although, the yield of biosurfactants between washed and unwashed carriers was not significantly different. The results suggested that cell washing process provided available spaces for nutrient transfer to the cells during reactivation process. This study showed that immobilized cells could be reused for long-term biosurfactant production, which could reduce the cost and operation time for new inoculum preparation and biosurfactant production. Nonetheless, immobilized *Weissella cibaria* PN3 cells should not be used for more than nine production cycles due to the decreasing biosurfactant yields and cell numbers. Similarly, Christovaa et al. (2013) found that polyethylene oxide (PEO)-immobilized *Pseudomonas aeruginosa* strain BN10 produced a rhamnolipid yield of 4.6 g/L per cycle until the 6th production cycle, and the yields decreased afterward. To improve the biosurfactant production process, it is possible to inoculate fresh bacterial cells during the reactivation step, which could increase the numbers of immobilized cells on the carrier.

In the 1-month period, nine biosurfactant production cycles could be carried out with an overall crude biosurfactant yield of 24.67 g/L (mixture of extracellular and cell-bound biosurfactants). The overall biosurfactant yields observed in this study are considerably higher than those in other repeated biosurfactant production studies using free cells. For example, Joy et al. (2019) reported a maximum overall rhamnolipid production of 19.35 g/L from *Achromobacter* sp. PS1 after five cycles of sequential fill and draw approach during 18-day period, and Bustos et al. (2018) showed that *Lactobacillus pentosus* biomass produced had an overall biosurfactant production of only 2.7 g/L after three cycles during a 45-h period. It is possible to apply immobilized *Weissella cibaria* PN3 cells in a scale-up system for long-term biosurfactant production. Moreover, biosurfactant production using complex agro-industrial wastes instead of high-cost medium components via solid state fermentation or submerged fermentation (Mnif and Ghribi, 2016) can further reduce biosurfactant production cost.

The extracellular and cell-bound biosurfactants were able to reduce surface tension comparable to other LAB biosurfactants and had similar CMC values (Table 1). For example, Sharma et al. (2014) showed that *L. helveticus* MRTL 91 reduced surface tension to 39.5 mN/m with a CMC of 2.5 g/L, while Ferreira et al. (2017) reported that extracellular biosurfactant from *L. paracasei* reduced surface tension to 25 mN/m with a CMC of 1.35 g/L. Cell-bound biosurfactant of *L. agilis* CCUG31450 reduced surface tension to 42.5 mN/m with a CMC of 7.5 g/L (Gudina et al., 2015), and lipoprotein derived from *Pediococcus dextrinicus* reduced surface tension to 39.0 mN/m (Ghasemi et al., 2019). Thus, these biosurfactants had good surface activity.

The extracellular and cell-bound biosurfactants had high emulsification activities with lavender and lemongrass oils. The results suggested that the biosurfactants were relatively hydrophilic because they interacted better with small molecular weight oils with hydrophilic structures. For petroleum oils, biosurfactants produced better emulsions with gasoline and diesel oil than BKC and ARL/AXL blend crude oil. This is because engine oils contain detergents, dispersants, friction modifiers, viscosity modifiers, anti-freeze agents, antioxidants, and others (Glos et al., 2014), which promote emulsification. Similarly, biosurfactant from *L. paracasei* performed 70 and 62.5% of EV with almond and essential oils, respectively (Ferreira et al., 2017), while *L. pentosus* biosurfactant gave 100% of EV with rosemary oil (Rodriguez-Lopez et al., 2018). Satpute et al. (2019) reported that *L. acidophilus* biosurfactant gave the highest EV value of 65% with n-decane followed by xylene (46%) and other hydrocarbons. Interestingly, the mixture of extracellular and cell-bound biosurfactants could emulsify palm, soybean and BKC crude oil, whereas a single biosurfactant did not. The results indicated that a synergistic effect occurred between these biosurfactants. Similarly, mixed rhamnolipid showed different emulsifying activities with liquid paraffin, kerosene and n-hexane when compared to mono-RLs and di-RLs (Zhou et al., 2019). The synergistic effect of mixed surfactants has been found to reduce surface tension and mixed micelle formation (Kumari et al., 2018).

The oil displacement efficiency of extracellular and cell-bound biosurfactants for BKC and ARL/AXL blend crude oils in this study was lower than that in the study of Khondee et al. (2015), who reported that lipopeptides from *Bacillus* sp. GY19 showed 100% oil displacement efficiency with diesel oil, followed by 76–84% of ARL/AXL blend crude oil. In addition, Rongsayamanont et al. (2017) reported that mixing lipopeptides with SDHS increased the efficiency of lipopeptides and showed 100% oil displacement efficiency with BKC and >90% oil displacement efficiency with an ARL/AXL blend. The low oil displacement efficiency of biosurfactants from *Weissella cibaria* PN3 could be due to the lower hydrophobicity than lipopeptides. To improve the oil displacement activity, the biosurfactants should be mixed with another hydrophobic biosurfactant to achieve a hydrophilic-lipophilic balance. In contrast to the emulsifying activity, the mixed biosurfactants did not show synergistic effects with oil displacement activity. This is because the mechanisms of oil displacement and emulsion formation are different. Oil displacement occurs when the interfacial tension between the water and oil phases is sufficiently reduced and overcomes the capillary force, which is related to interfacial tension between the aqueous and oil phases and not emulsion formation (De Almeida et al., 2016).

Biosurfactants from *Weissella cibaria* PN3 at concentrations ranging from 10 to 16 mg/mL showed antimicrobial activity toward some bacteria and yeast but not filamentous fungi. The antimicrobial activities of these biosurfactants were comparable to those of other LAB strains, especially their cell-bound biosurfactants. For example, *L. plantarum* CFR 2194 cell-bound biosurfactant at 25 mg/mL inhibited the growth of *Staphylococcus aureus* F772 (Madhu and Prapulla, 2014); *L. pentosus* cell-bound biosurfactant at 50 mg/mL had significant antimicrobial activity against *Pseudomonas aeruginosa*, *Streptococcus agalactiae*, *Staphylococcus aureus*, *Escherichia coli*, *Streptococcus pyogenes*, and *Candida albicans* (Vecino et al., 2018); and cell-bound biosurfactants of *Pediococcus acidilactici* and *L. plantarum* showed antimicrobial activity against *S. aureus* CMCC 26003 at >100 mg/mL (Yan et al., 2019). In this study, the cell-bound biosurfactant had higher antimicrobial activity than the extracellular biosurfactant. Biosurfactants from LAB strains also show antiadhesive and antibiofilm properties (Madhu and Prapulla, 2014; Gudina et al., 2015; Hajfarajollah et al., 2018; Yan et al., 2019); thus, it is interesting to explore these biosurfactants further.

Only a few studies have investigated the characteristics of biosurfactants from LAB. Biosurfactants from various LAB were found to be glycolipids (Sharma et al., 2014), glycoproteins (Madhu and Prapulla, 2014; Gudina et al., 2015), and lipoproteins (Ghasemi et al., 2019). The different biosurfactant characteristics depend on the variability of biosurfactant metabolism by the bacterial strain and carbon source used (Mouafo et al., 2018). Based on chemical composition, the biosurfactants from *Weissella cibaria* PN3 using soybean oil as substrate were glycolipids, and the different FTIR chromatograms indicated that the extracellular and cell-bound glycolipid congeners were different. The hydrophilic heads of the extracellular and cell-bound biosurfactants should be further investigated via nuclear magnetic resonance (NMR) spectroscopy to confirm biosurfactant types.

## Conclusion

Immobilized *Weissella cibaria* PN3 cells could be reused for up to nine cycles of glycolipid biosurfactant production. This biosurfactant production process is relatively cheap given that two types of biosurfactants were produced simultaneously and no new bacterial inoculum was required. The extracellular and cell-bound biosurfactants showed different surface activities, oil displacement, emulsifying activities and antimicrobial activities. Thus, they might be applied separately or as a mixture in various products, such as cleaning agents, food-grade emulsions and cosmetics. The optimization of production medium and utilization of agricultural wastes as substrate should be explored to further reduce production cost. Further, this biosurfactant production process using immobilized cells could be applied to other LAB strains.

## Data Availability Statement

All datasets generated for this study are included in the article/Supplementary Material.

## Author Contributions

TS conducted most of the experiments, interpreted the results, and wrote the manuscript. NK, WR, and C-YC involved in the characterization of biosurfactants. PN assisted in bacterial cultivation and biosurfactant analysis. EL conceived the overall project, acquired the funding, performed the manuscript editing and final improvement. All authors contributed to the preparation of manuscript.

## Conflict of Interest

The authors declare that the research was conducted in the absence of any commercial or financial relationships that could be construed as a potential conflict of interest.
