# Supplementary material for: Reuse of Immobilized Weissella cibaria PN3 for Long-Term Production of Both Extracellular and Cell-Bound Glycolipid Biosurfactants
Source: Front Bioeng Biotechnol. 2020 Jul 3;8:751. doi: 10.3389/fbioe.2020.00751 (PMC7347796; doi:10.3389/fbioe.2020.00751)
Supplement: Supplementary file 1 [file Data_Sheet_1.pdf]

Supplementary Material

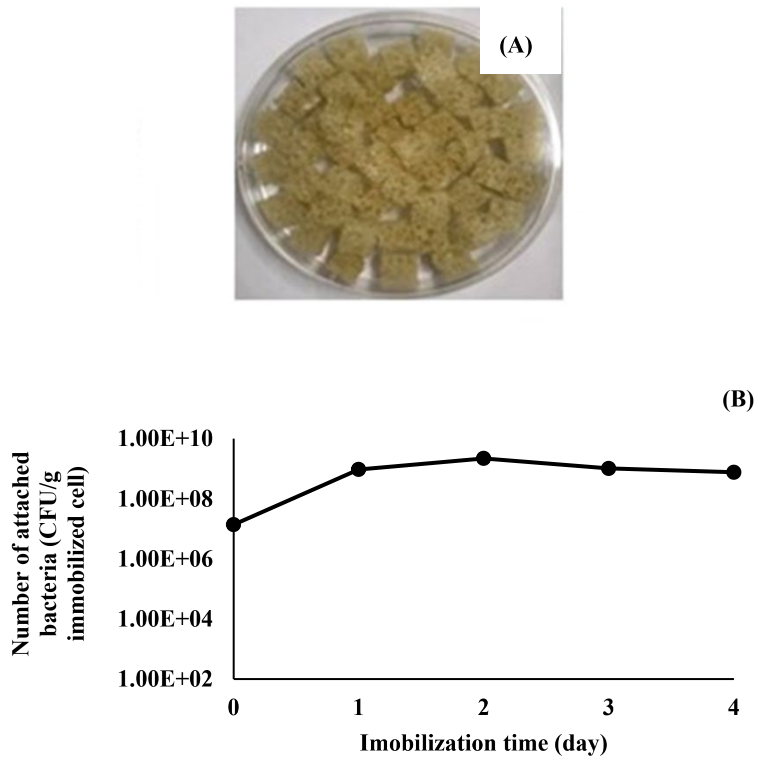

Supplementary Figure 1. The characteristics of aquaporousgel after biosurfactant production (A). The numbers of attached bacteria during cell immobilization were monitored (B).

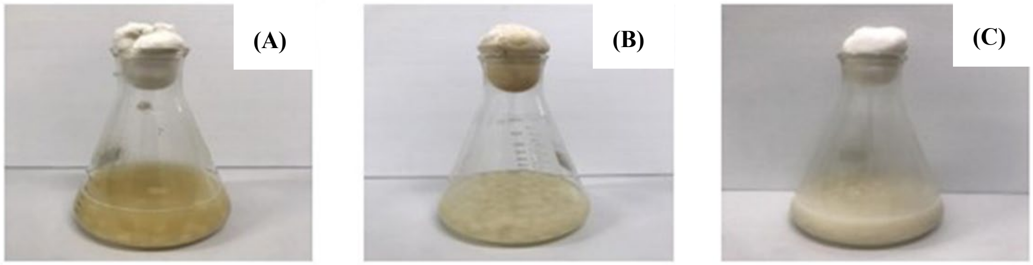

Supplementary Figure 2. Immobilized *Weissella cibaria* PN3 in LB broth during immobilization process (A) and in basal medium supplemented with 2% (v/v) soybean oil before (B) and after biosurfactant production (C).

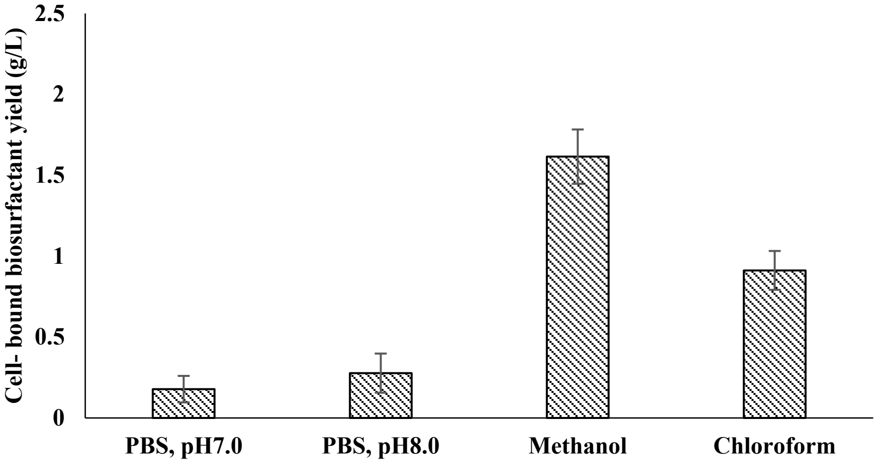

Supplementary Figure 3. The yields of cell-bound biosurfactant after resuspending the cell pellets in PBS, methanol and chloroform.

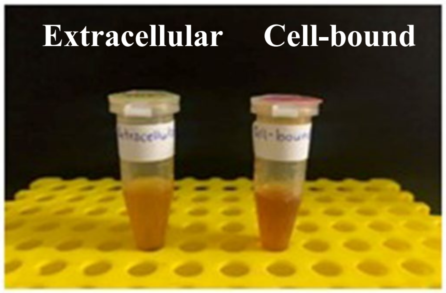

Supplementary Figure 4. Crude extracellular and cell-bound biosurfactant from immobilized-*Weissella cibaria* PN3

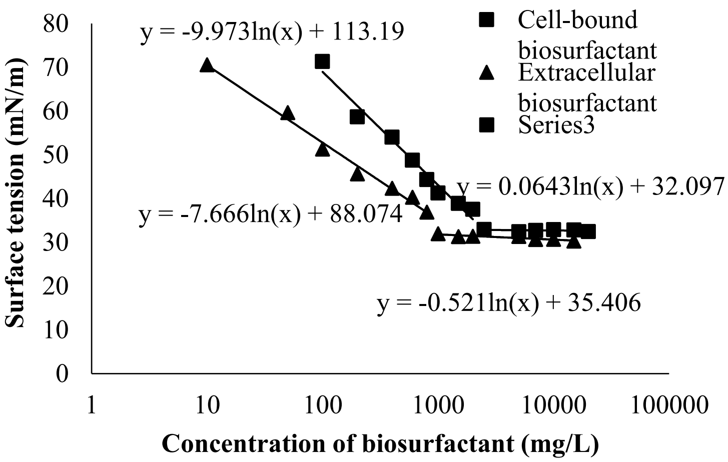

Supplementary Figure 5. Evaluation of critical micelle concentration (CMC) of crude extracellular and cell-bound biosurfactants.

Supplementary Table1. Surface tension of crude extracellular and cell-bound biosurfactants at the CMC value.

Biosurfactant types	Equation	Surface tension	Average surface tension (mN/m)
Extracellular biosurfactant	Y = -7.621ln(x) +87.970	31.32	31.32±0.08
	Y = -7.710ln(x) + 88.179	31.41	
	Y = -7.66ln(x) +88.065	31.25	
Cell-bound biosurfactant	Y = -9.983ln(x) +112.992	32.58	32.59±0.03
	Y = -9.973ln(x) + 113.194	32.62	
	Y = -9.965 ln (x) + 112.890	32.56	

*Surface tension of PBS was 68.32±0.25 mN/m

(A)

(B)

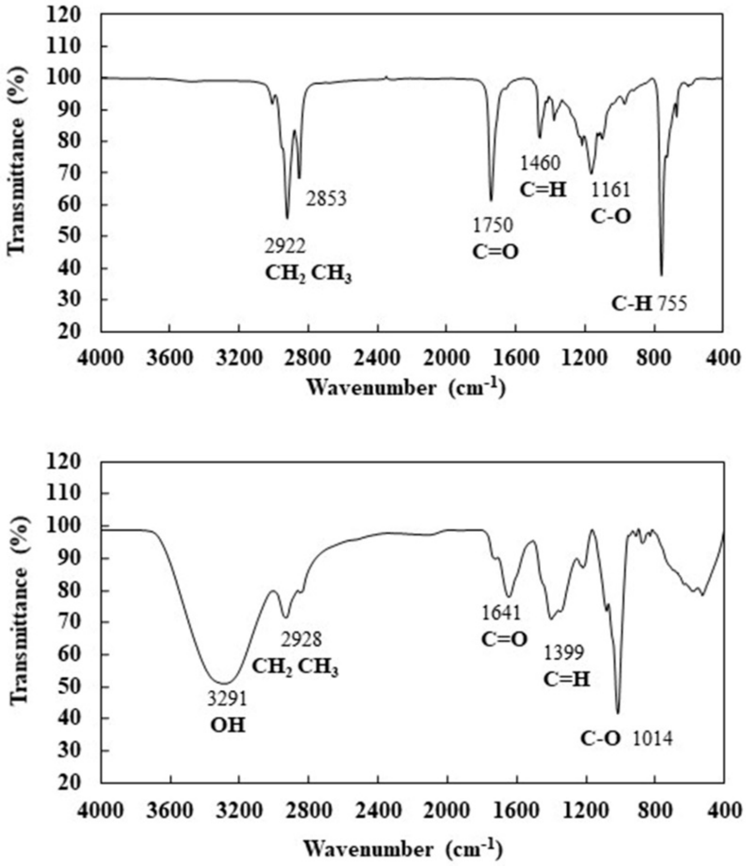

Supplementary Figure 6. FTIR spectrum of crude extracellular (a) and cell-bound biosurfactant (b) from *Weissella cibaria* PN3.
